# P-1866. Polymorphonuclear Myeloid-Derived Suppressor Cells regulates immune reconstitution during HIV infection through PD-L1 and TGF-β signaling

**DOI:** 10.1093/ofid/ofae631.2027

**Published:** 2025-01-29

**Authors:** Huan Xia

**Affiliations:** Tianjin Second People's Hospital, Tianjin, Tianjin, China

## Abstract

**Background:**

Although myeloid-derived suppressor cells (MDSCs) are widely recognized for their immunoinhibitory effect in a variety of pathological conditions, their function during human immunodeficiency virus (HIV) infection and the onset of inadequate immune reconstitution remains elusive.

PMN-MDSCs inhibit autologous CD4+ T-cell proliferation and cytokines (IL-2, TNF-α, and IFN-γ) production in HIV INRs.

**Figure:**
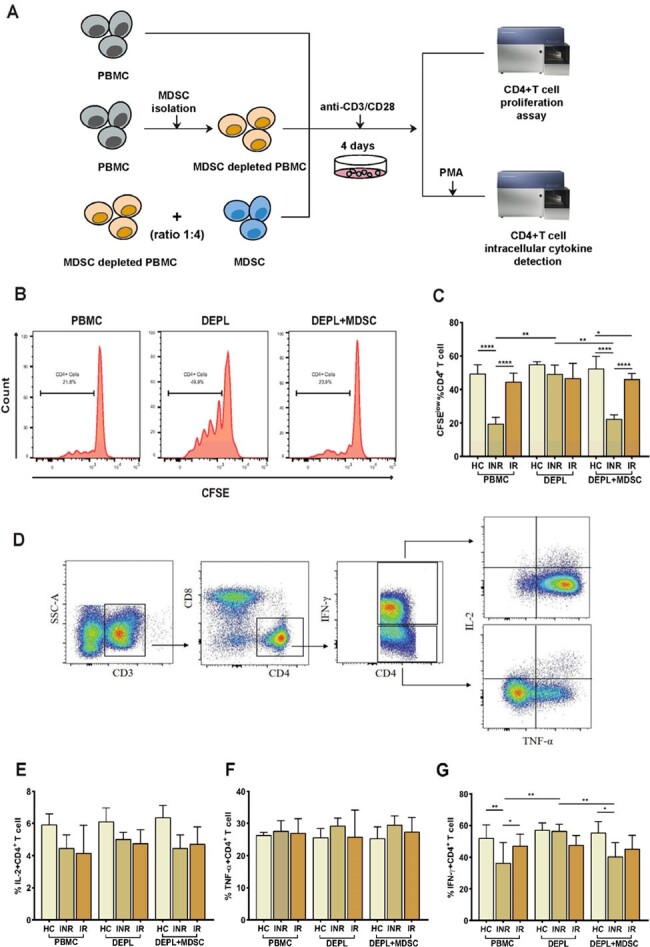

**Methods:**

We conducted a cross-sectional study in which 30 healthy controls (HC) and 62 HIV-1-infected subjects [31 immunological non-responders (INRs) and 31 immunological responders (IRs)] were selected. After two years of antiretroviral therapy, INRs and IRs were designated as patients with CD4+ T-cell counts < 350 cells/μL and >500 cells/μL, respectively. The proportion of MDSCs was measured and their relationship with HIV disease progression was studied. Specifically, using flow cytometry and real-time PCR, immune regulatory molecules (including programmed death-ligand 1 [PD-L1], arginase 1, inducible nitric oxide synthase, interleukin 10, transforming growth factor beta [TGF-β], and indoleamine 2,3-dioxygenase) that are relevant for MDSCs activity were quantified. Furthermore, we investigated the impact of the blockade of PD-L1 and/or TGF-β signaling on MDSCs and their effects on CD4+ T cells using *in vitro* functional experiments.

PD-L1 and TGF-β mediate the suppressive effect of PMN-MDSCs on CD4+ T-cells response.

**Figure:**
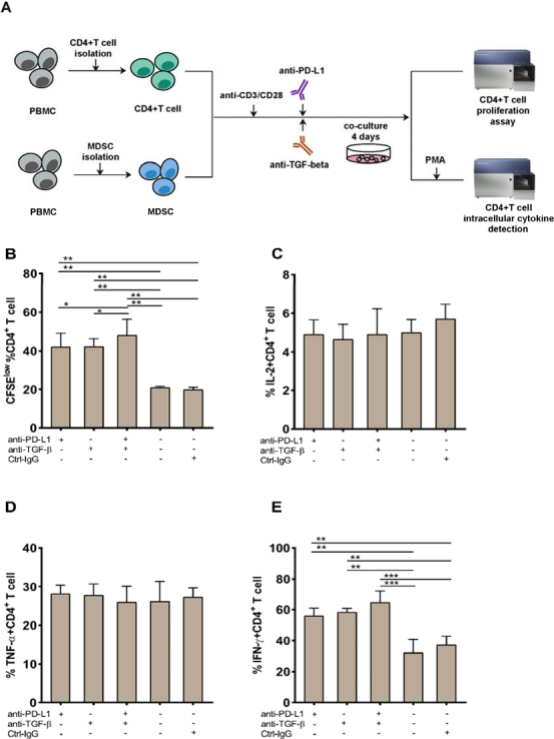

**Results:**

We found that polymorphonuclear MDSCs (PMN-MDSCs) are more abundant and negatively correlated with CD4+ T-cell counts in HIV-infected individuals. PMN-MDSCs suppress CD4+ T-cell proliferation and interferon-γ (IFN-γ) production in INRs. Furthermore, correlations were found between PD-L1 expression on PMN-MDSCs and PD-1+ CD4+ T-cells. TGF-β expression on PMN-MDSCs was likewise enhanced in INRs. Importantly, inhibiting both PD-L1 and TGF-β signaling had a synergistic impact on restoring CD4+ T-cell activity *in vitro*.

**Conclusion:**

Our findings reveal that the expansion of PMN-MDSCs decreased CD4+ T-cell proliferation and IFN-γ secretion in HIV infection through PD-L1 and TGF-β mediated pathways. Interventions that target both PD-L1 and TGF-β pathways may be novel strategies for enhancing immune reconstitution in INRs.

**Disclosures:**

All Authors: No reported disclosures

